# Supplementary figures and images for: gJLS2: an R package for generalized joint location and scale analysis in X-inclusive genome-wide association studies
Source: G3 (Bethesda). 2022 Feb 24;12(4):jkac049. doi: 10.1093/g3journal/jkac049 (PMC8982384; doi:10.1093/g3journal/jkac049)

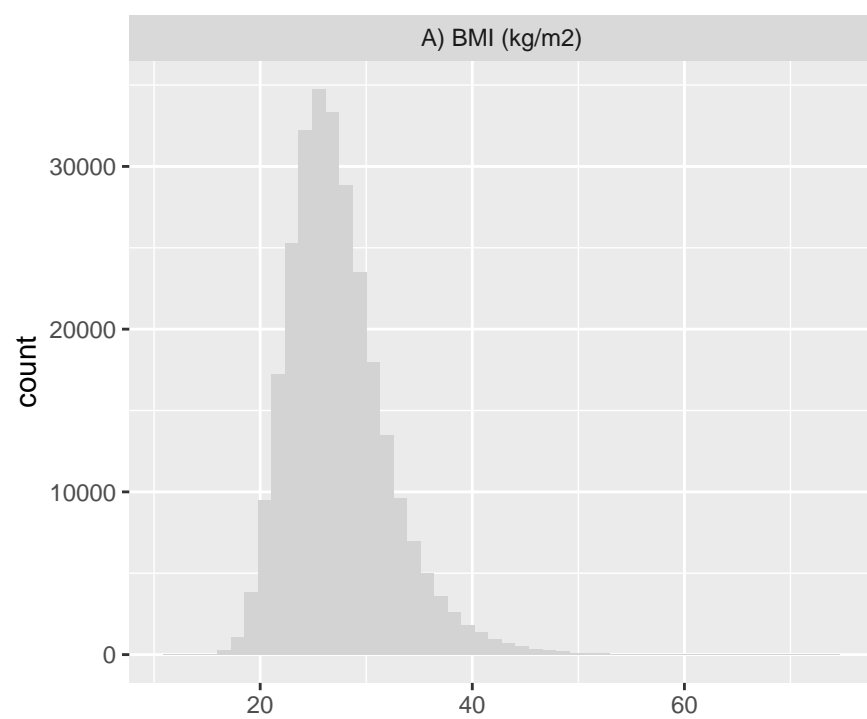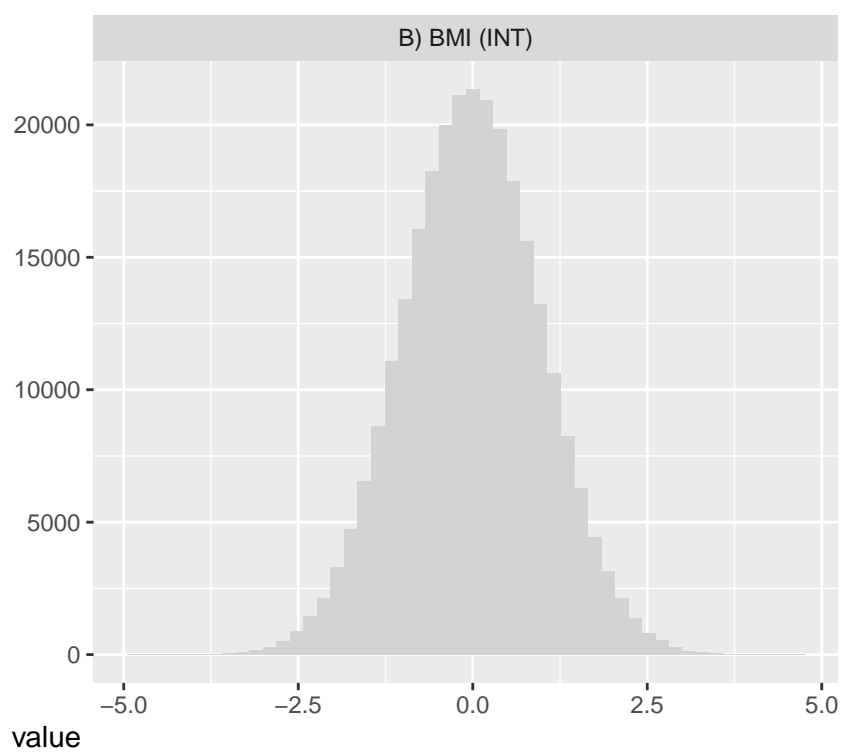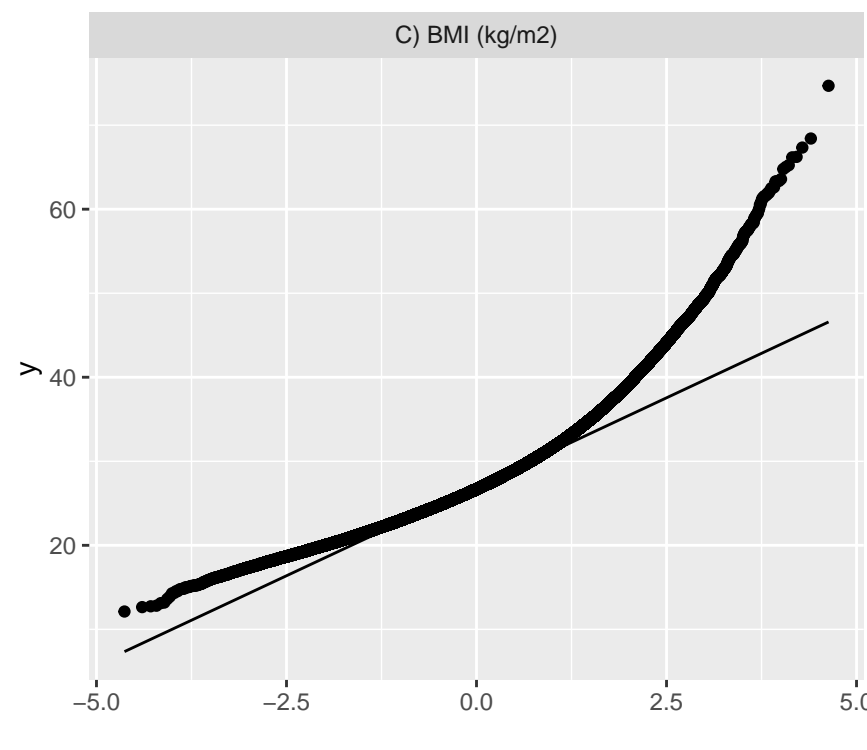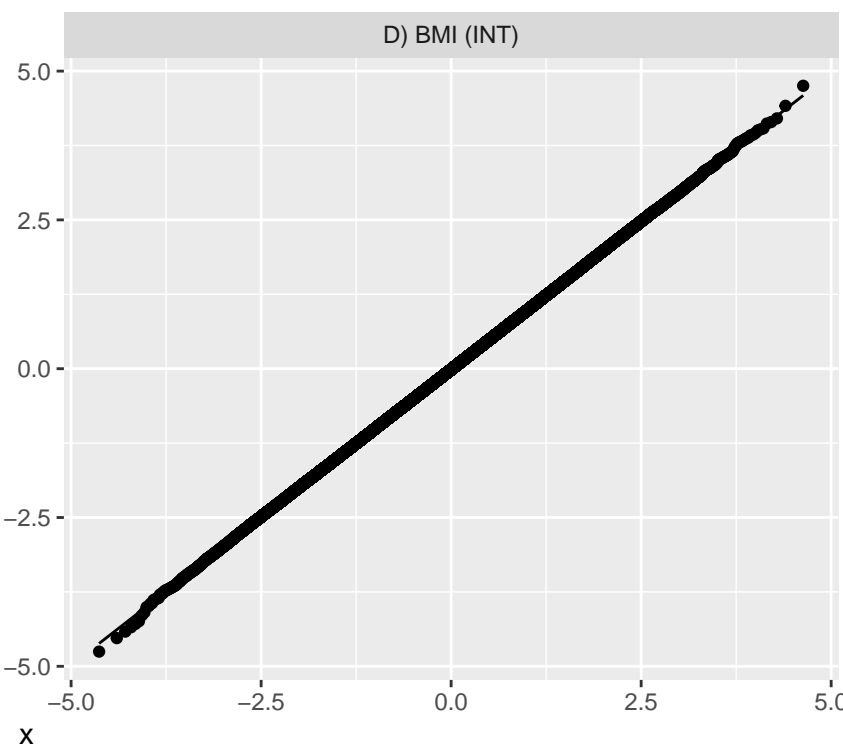

Supplement: jkac049_Supplementary_Data [file jkac049_supplementary_data.zip › Suppl/Supp.Figure_1_G3-2022-403216.pdf]

A)

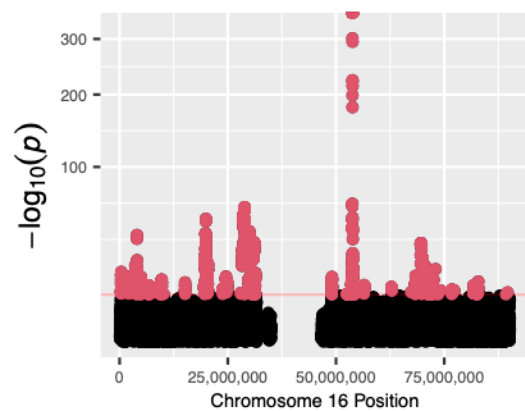

B)

Location

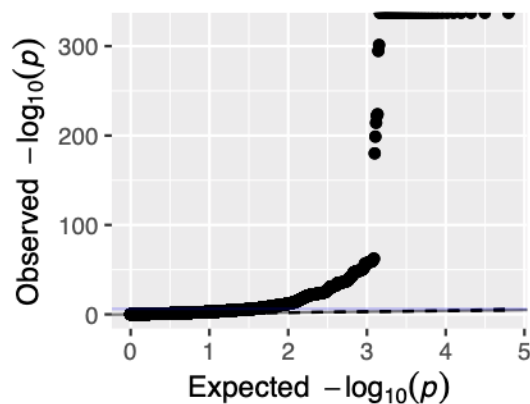

C)

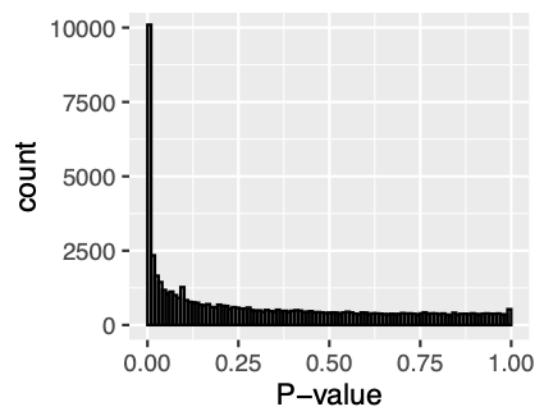

D)

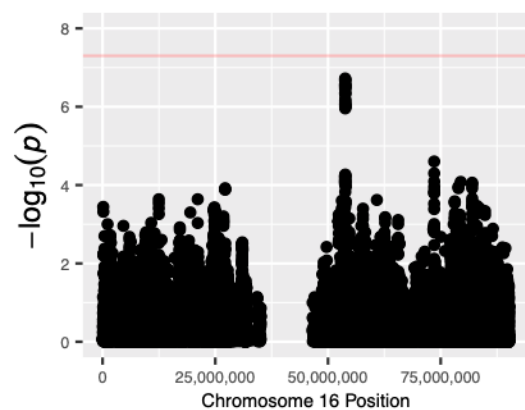

E)

Scale

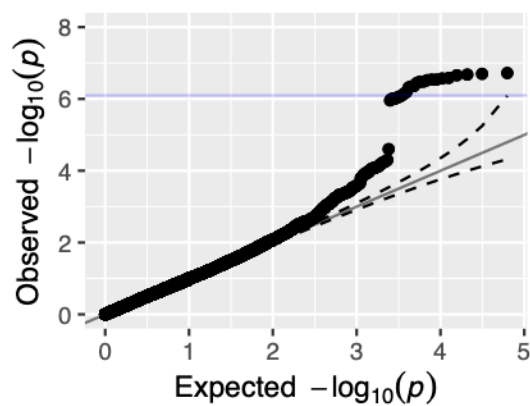

F)

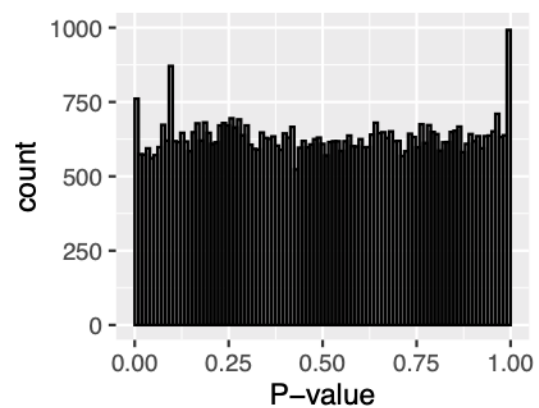

G)

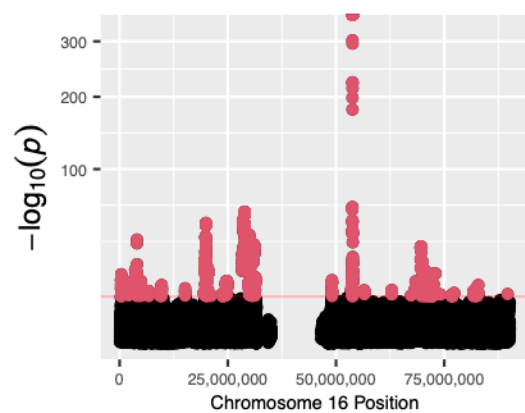

H)

gJLS

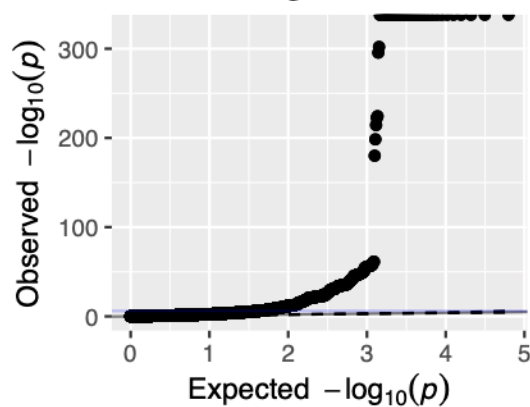

I)

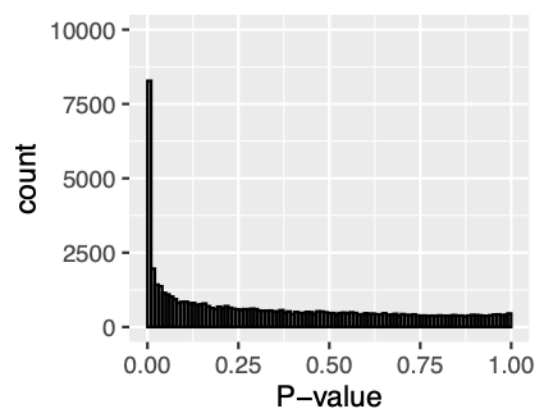

Supplement: jkac049_Supplementary_Data [file jkac049_supplementary_data.zip › Suppl/Supp.Figure_10_G3-2022-403216.pdf]

A)

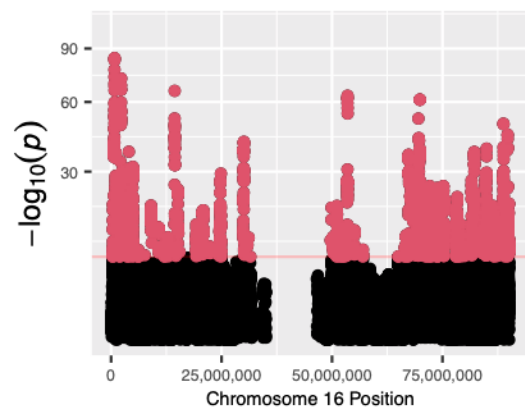B)  
Location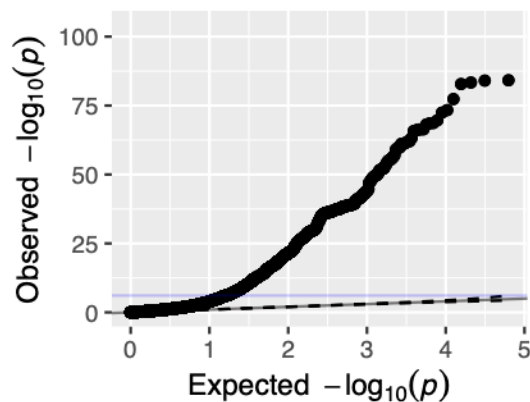

C)

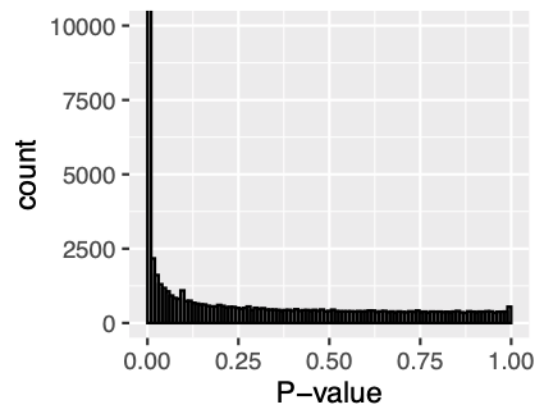

D)

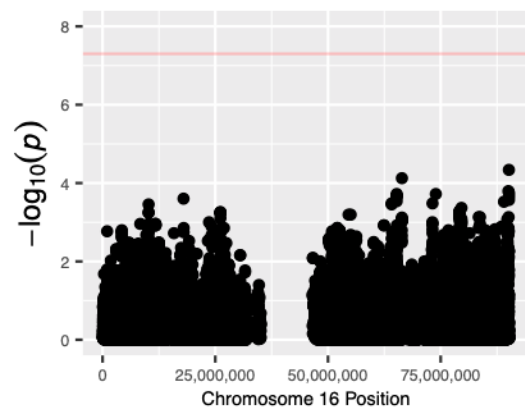E)  
Scale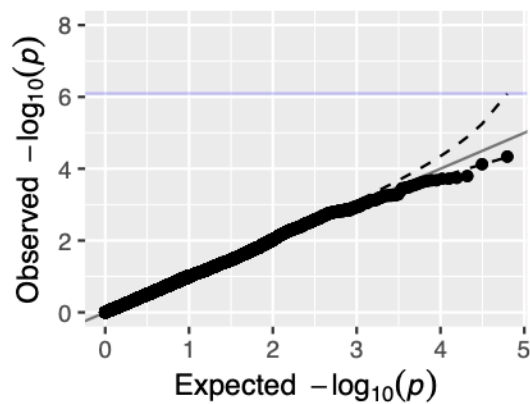

F)

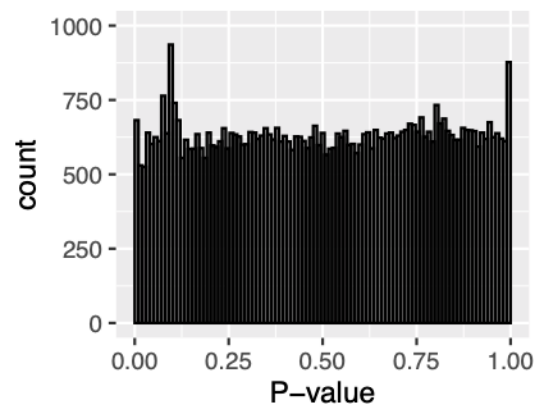

G)

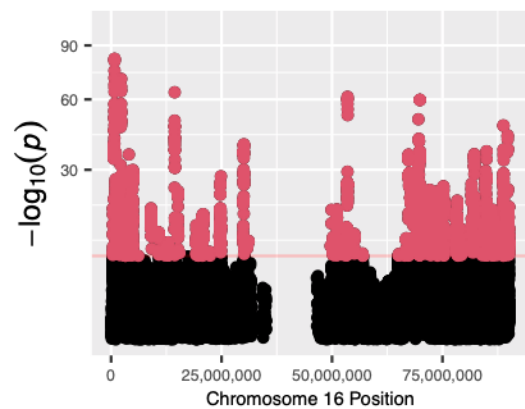H)  
gJLS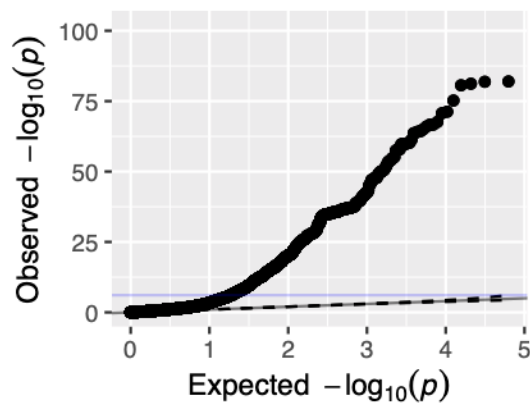

I)

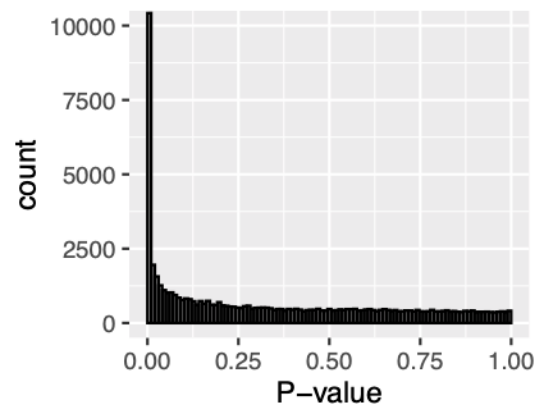

Supplement: jkac049_Supplementary_Data [file jkac049_supplementary_data.zip › Suppl/Supp.Figure_11_G3-2022-403216.pdf]

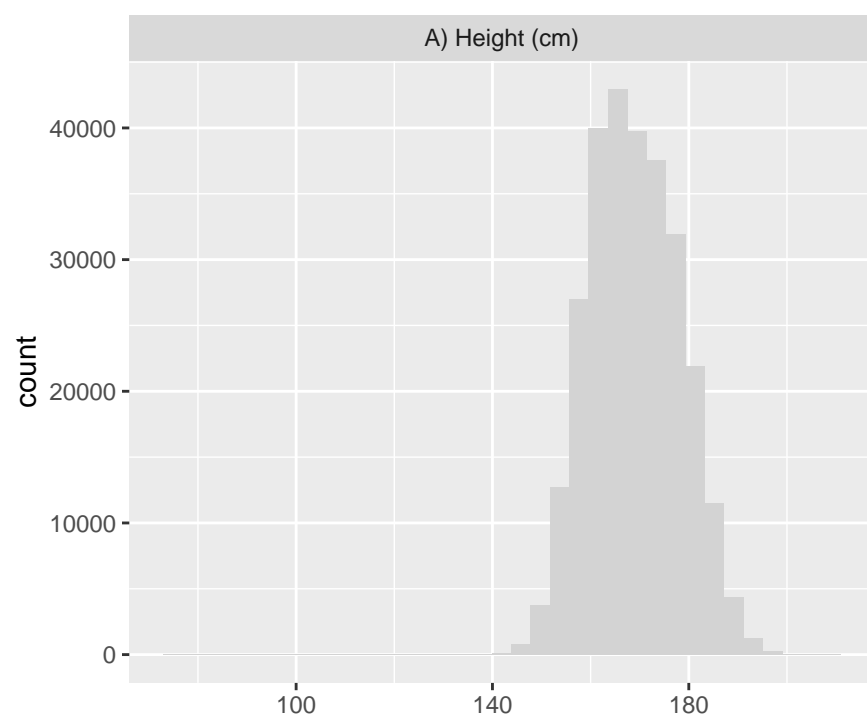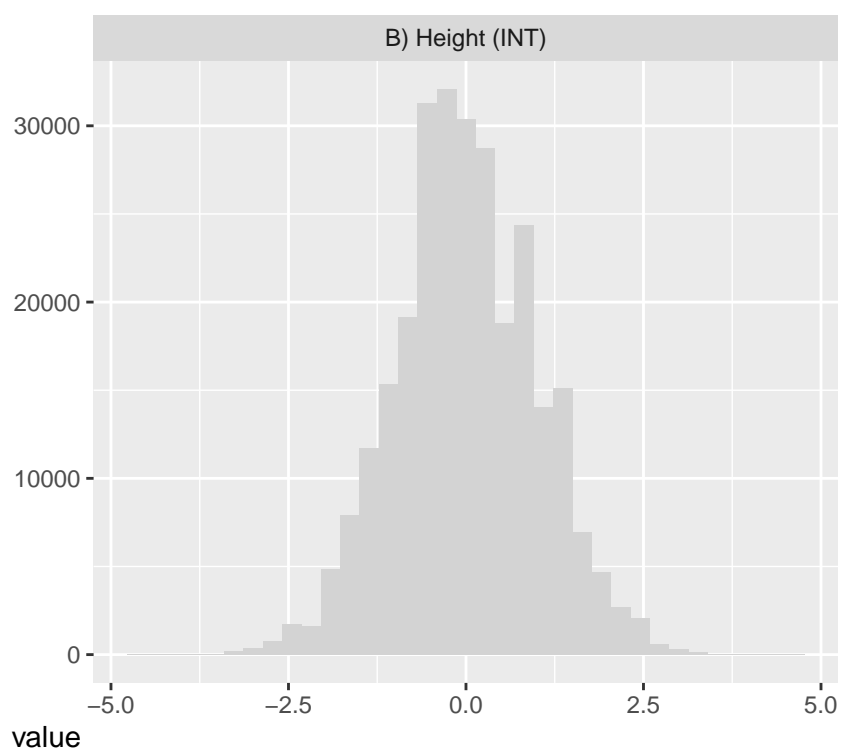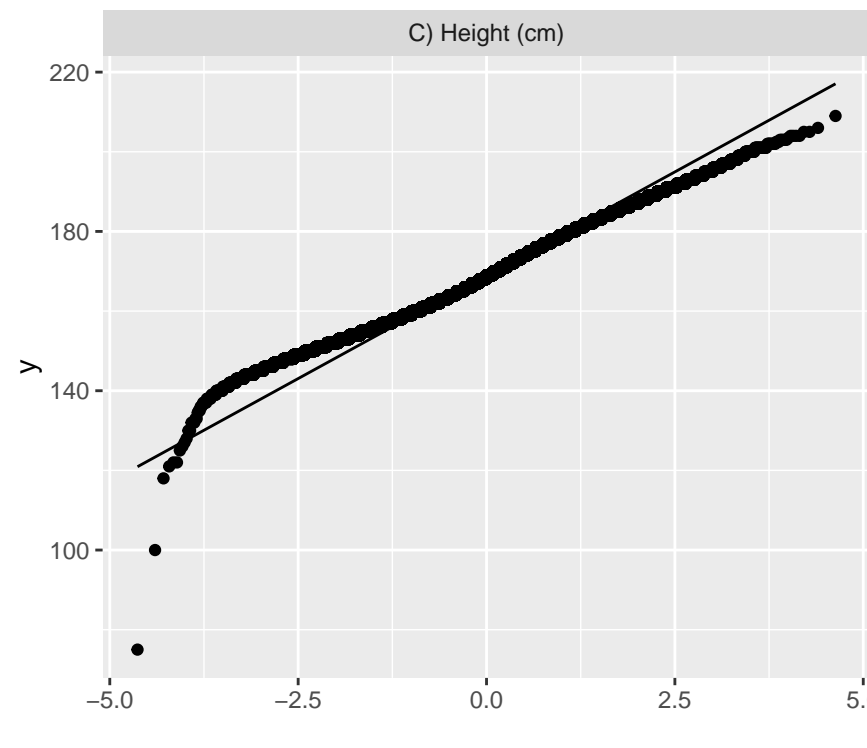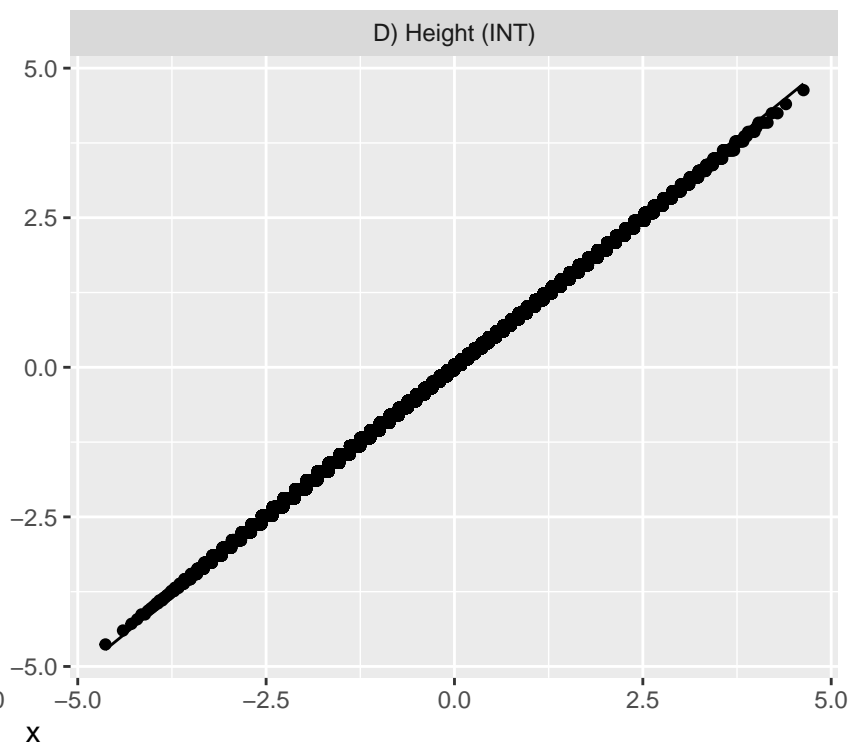

Supplement: jkac049_Supplementary_Data [file jkac049_supplementary_data.zip › Suppl/Supp.Figure_2_G3-2022-403216.pdf]

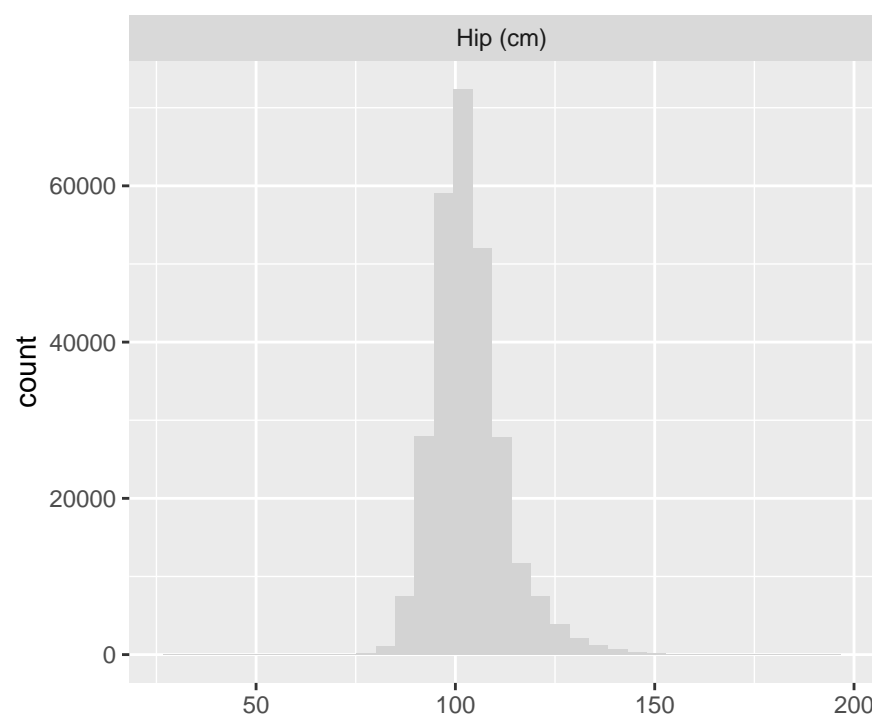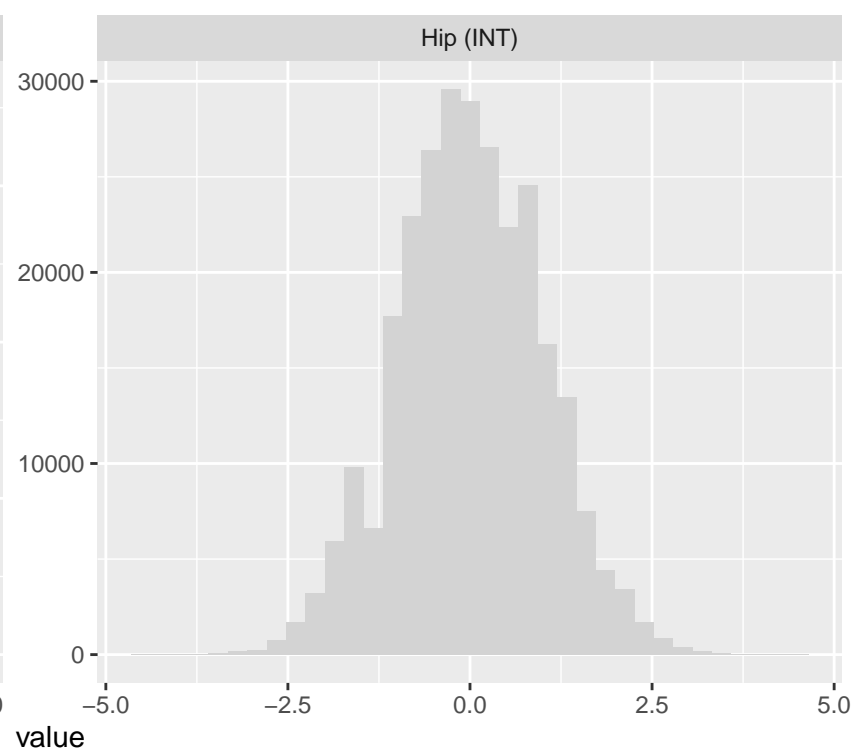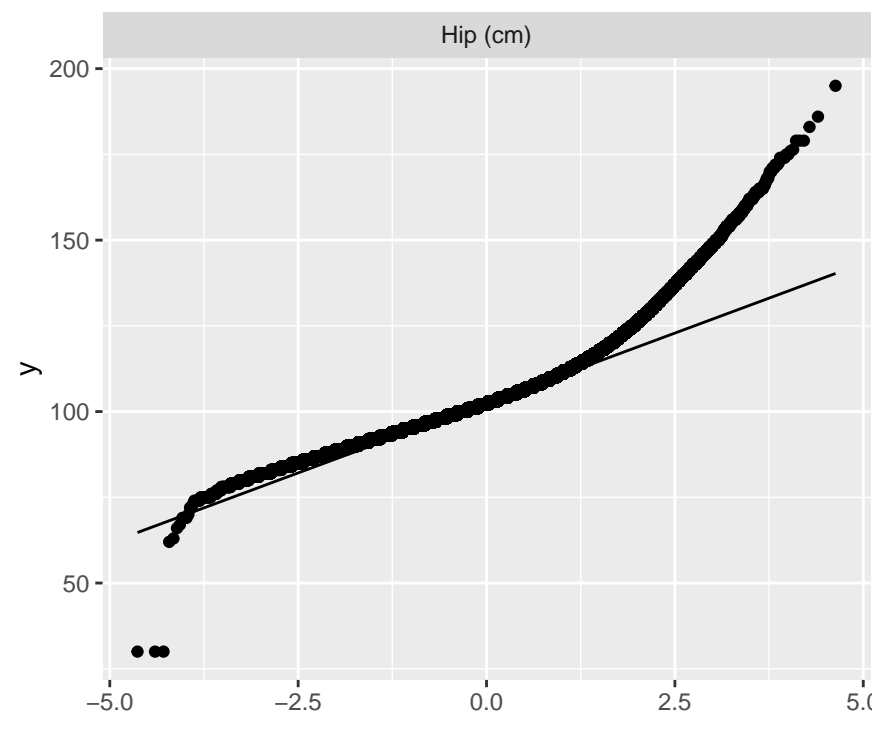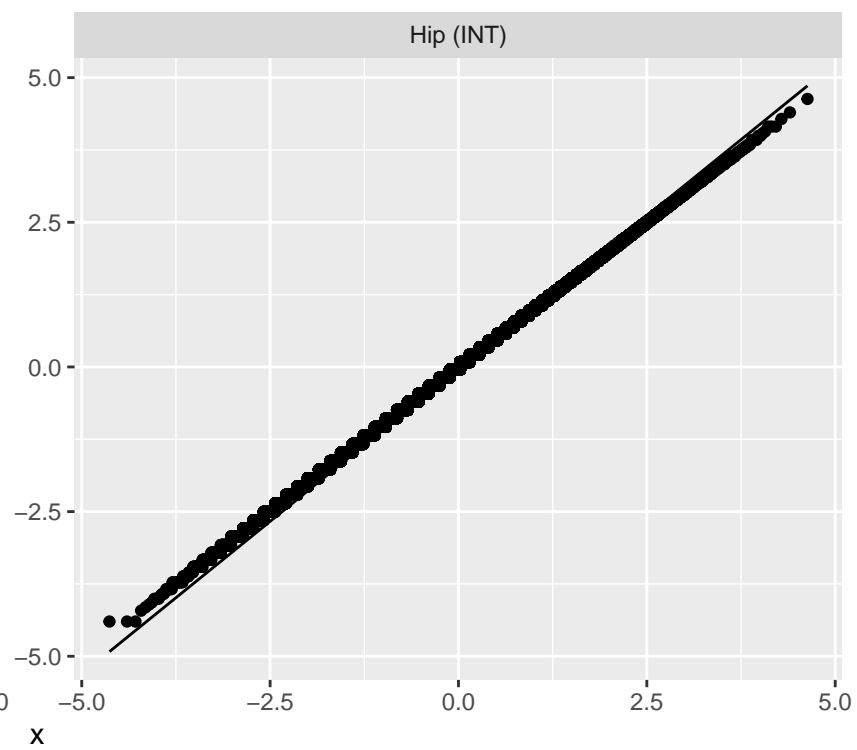

Supplement: jkac049_Supplementary_Data [file jkac049_supplementary_data.zip › Suppl/Supp.Figure_3_G3-2022-403216.pdf]

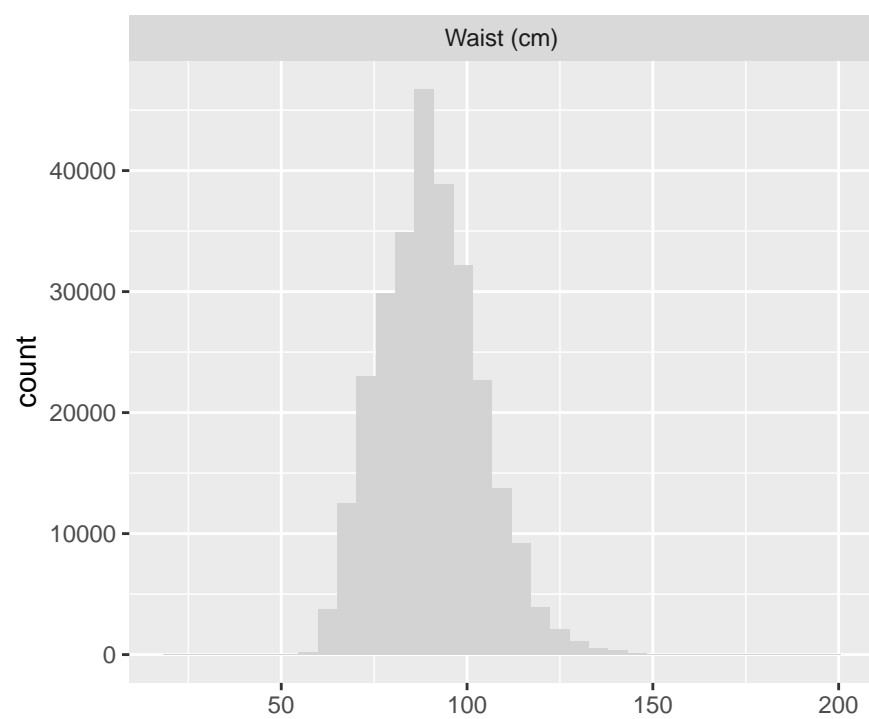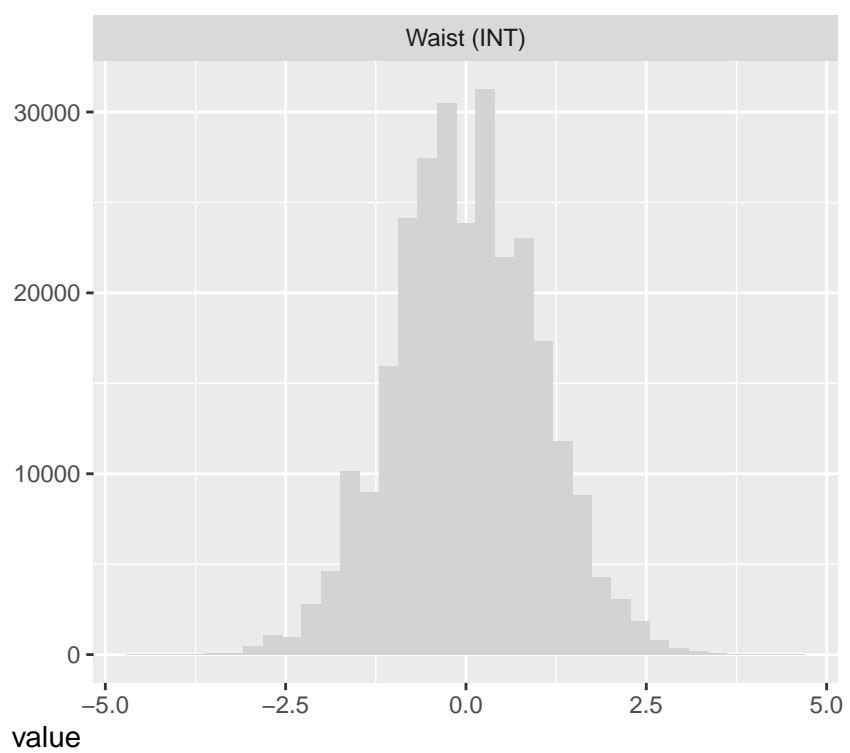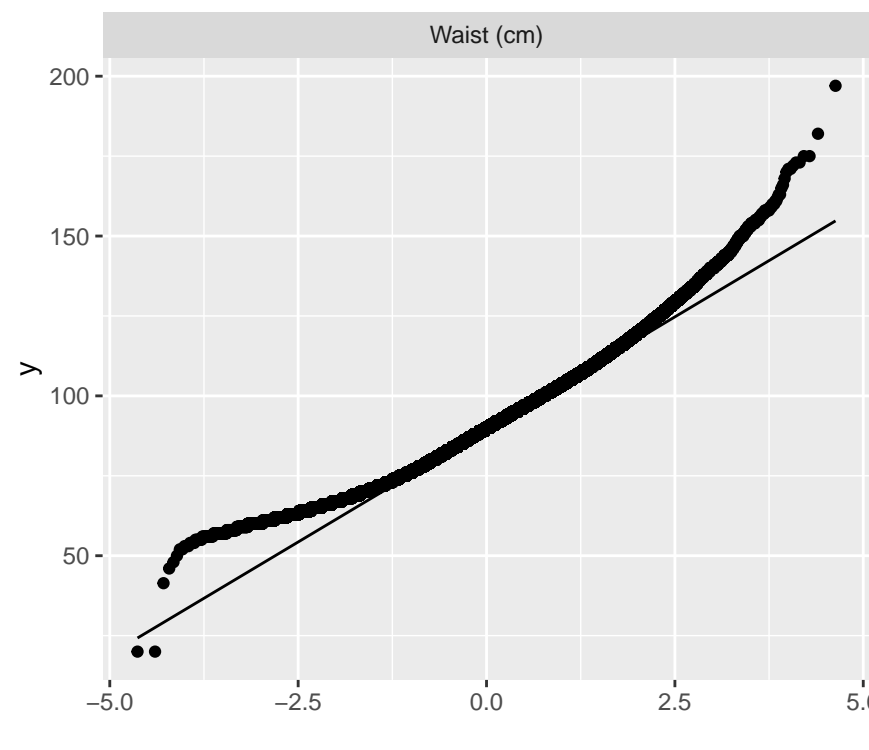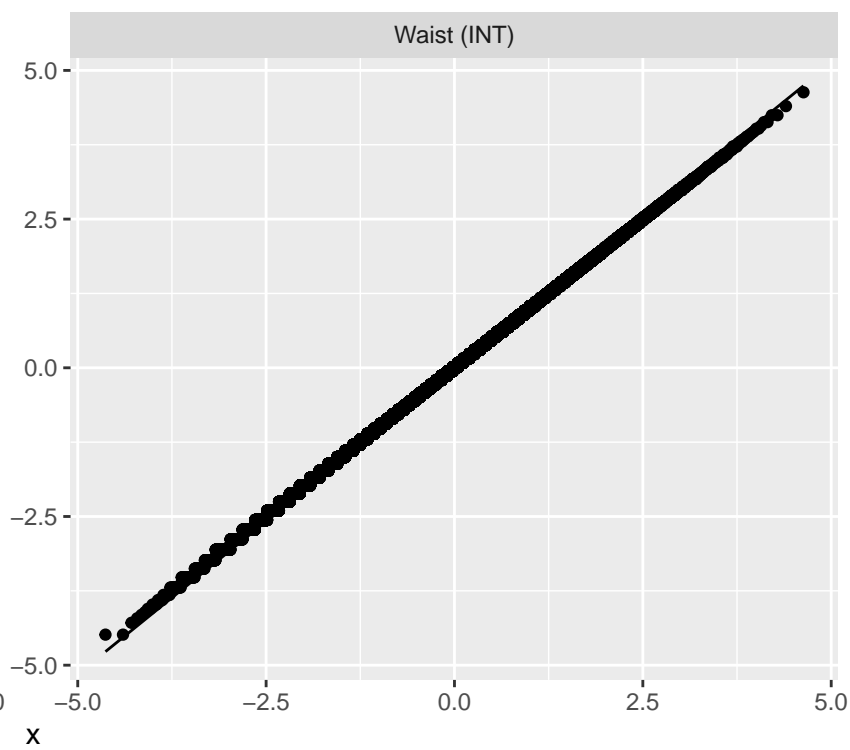

Supplement: jkac049_Supplementary_Data [file jkac049_supplementary_data.zip › Suppl/Supp.Figure_4_G3-2022-403216.pdf]

Female vs Males  
Allele Frequency of 15,179 X-chr SNPs

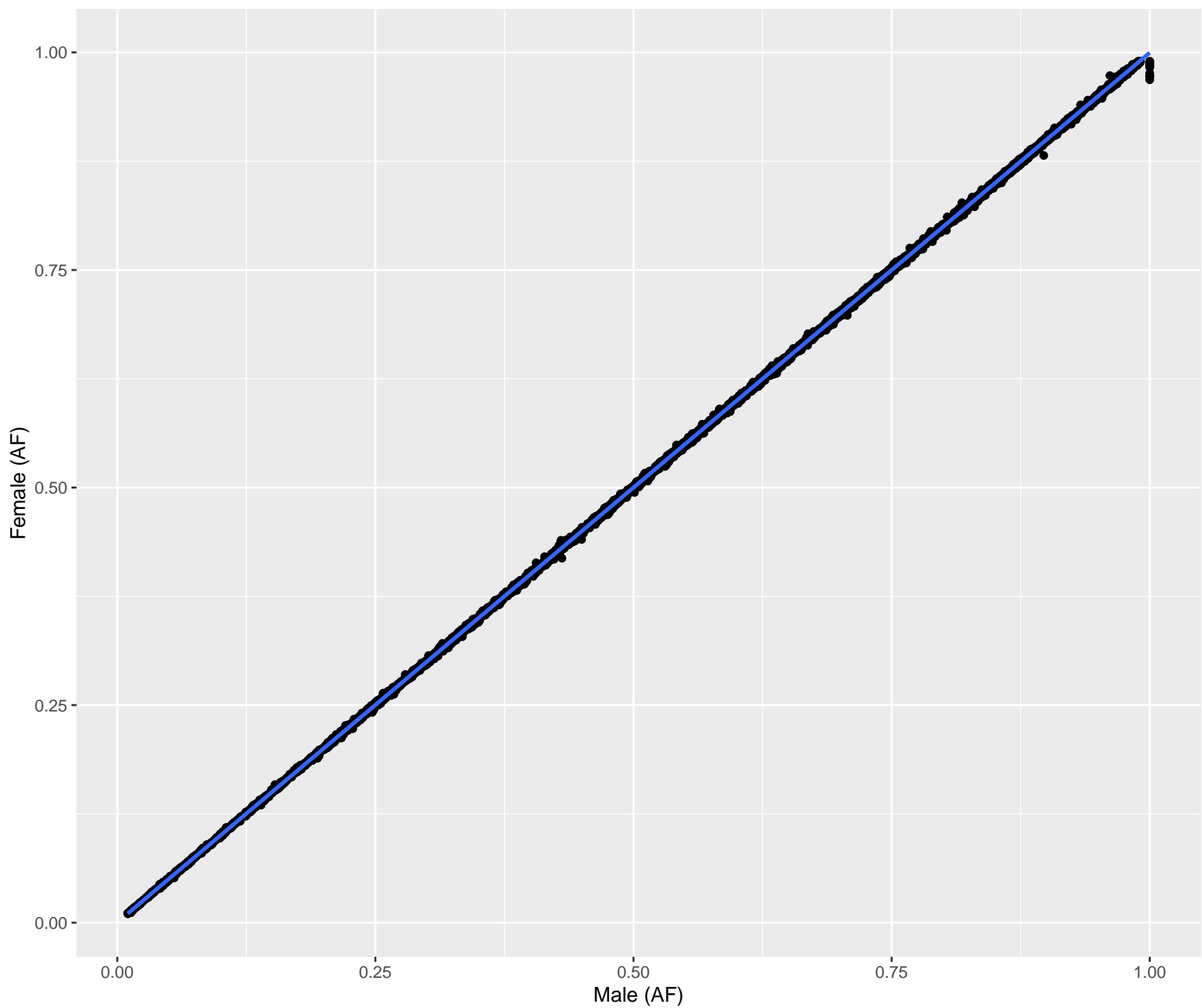

Supplement: jkac049_Supplementary_Data [file jkac049_supplementary_data.zip › Suppl/Supp.Figure_5_G3-2022-403216.pdf]

A)

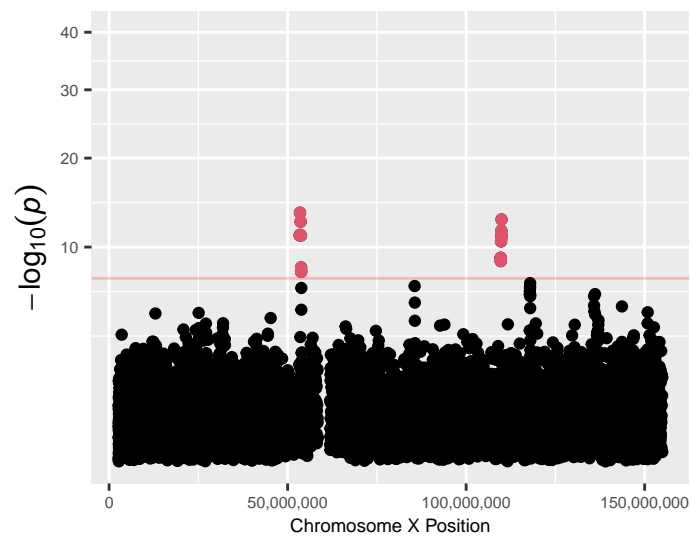B)  
Location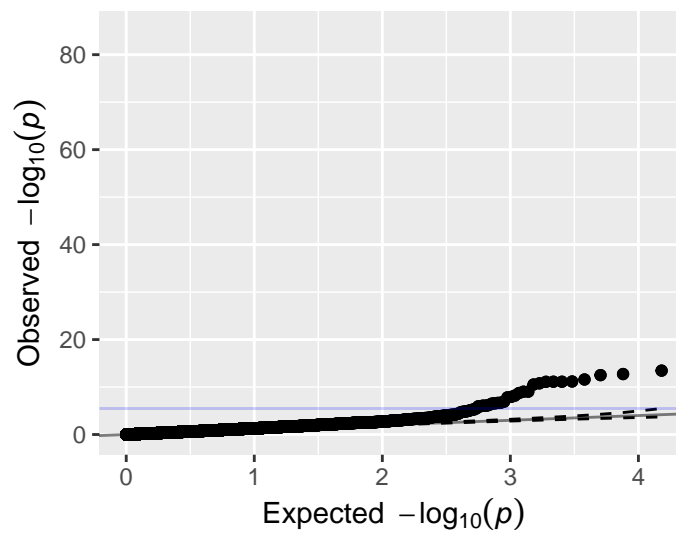

C)

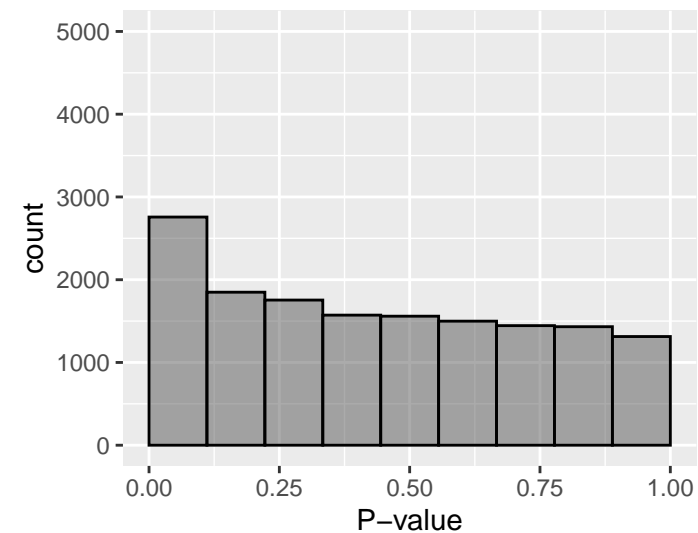

D)

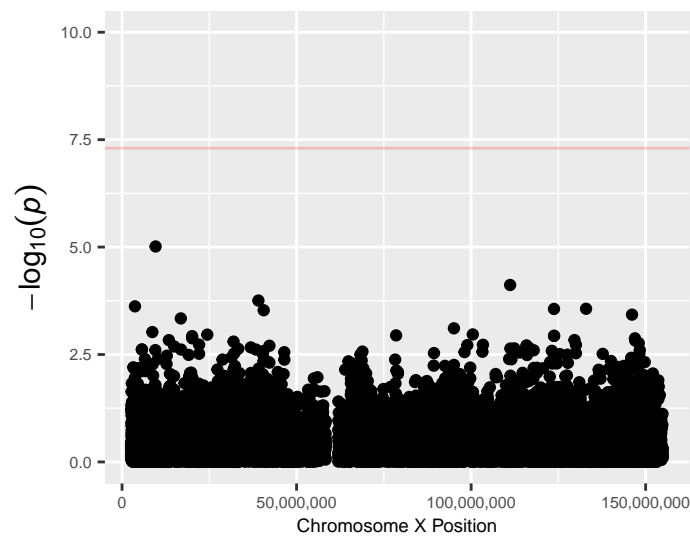E)  
Scale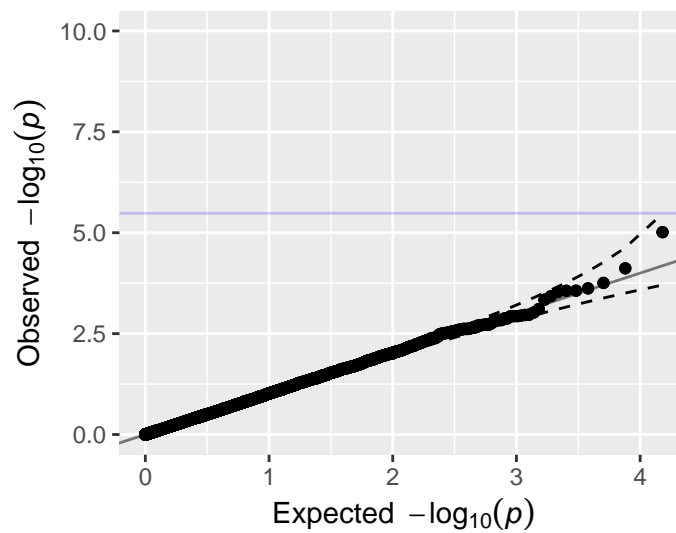

F)

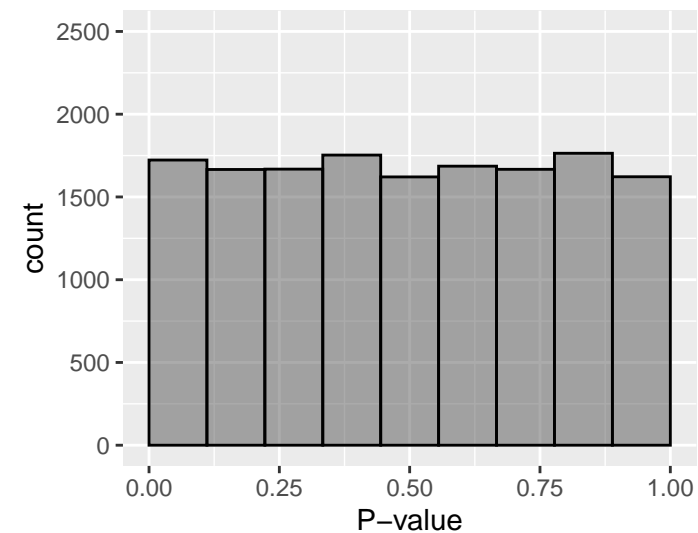

G)

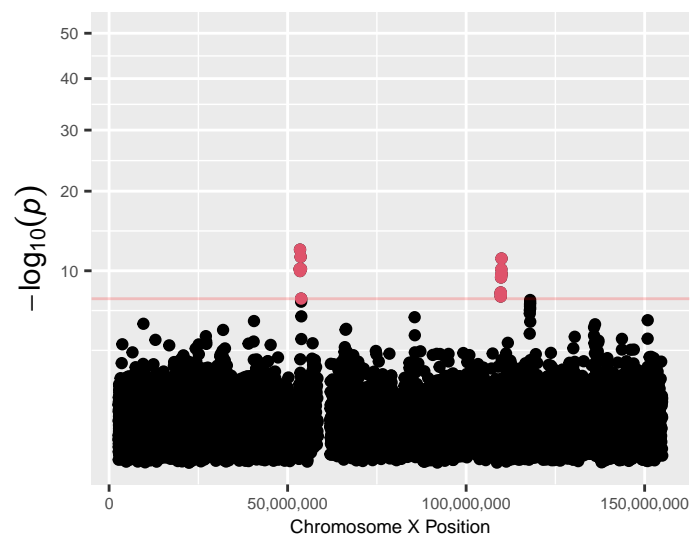H)  
gJLS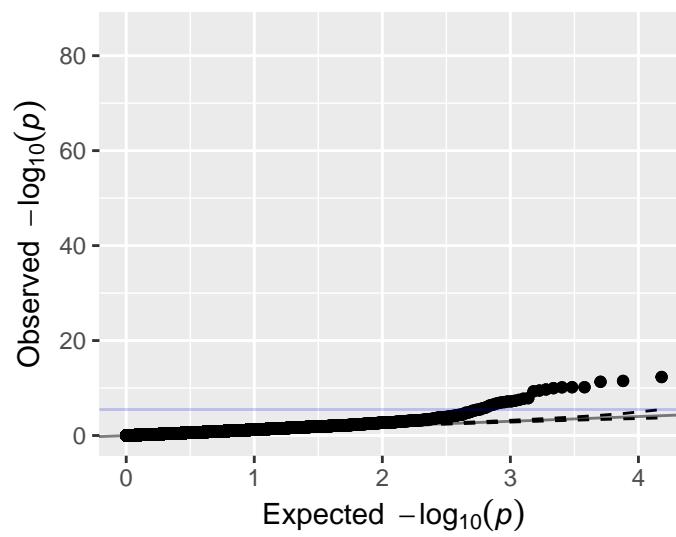

I)

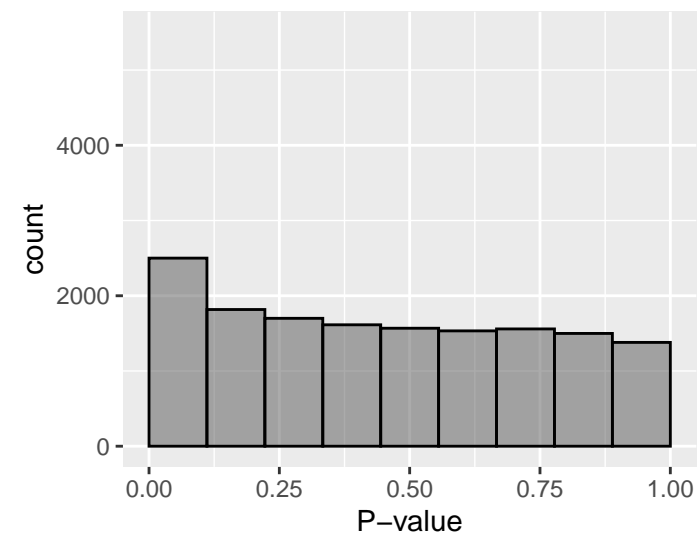

Supplement: jkac049_Supplementary_Data [file jkac049_supplementary_data.zip › Suppl/Supp.Figure_6_G3-2022-403216.pdf]

A)

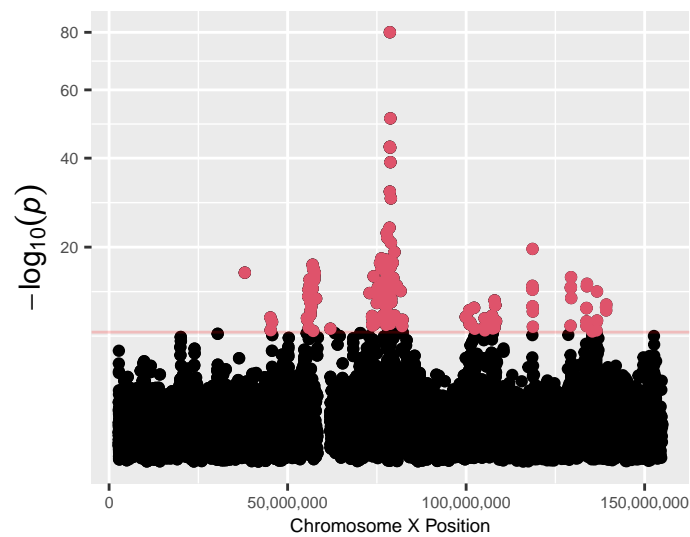B)  
Location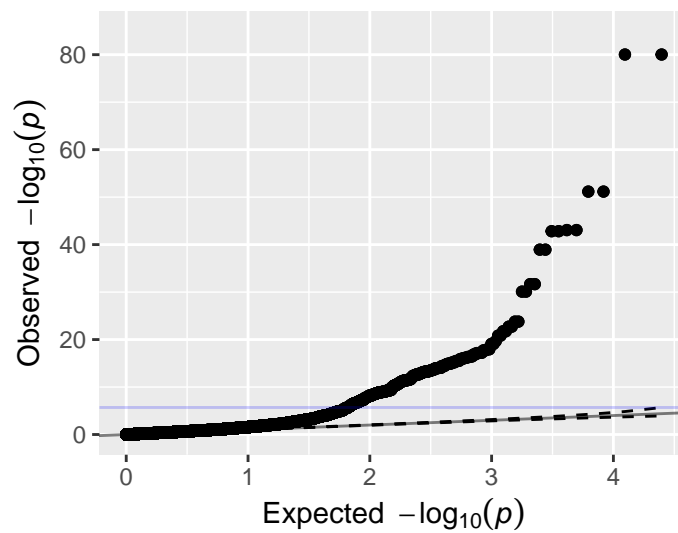

C)

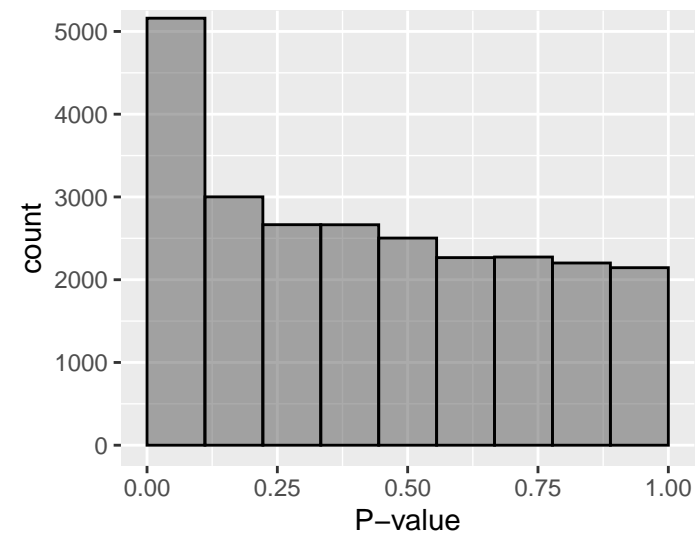

D)

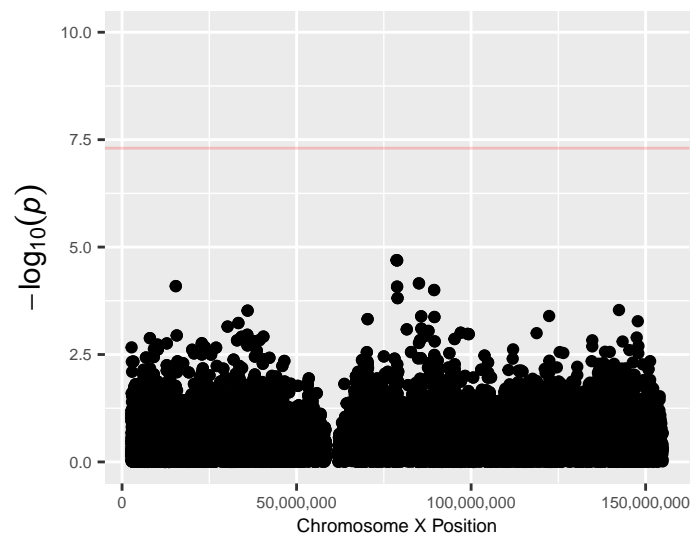E)  
Scale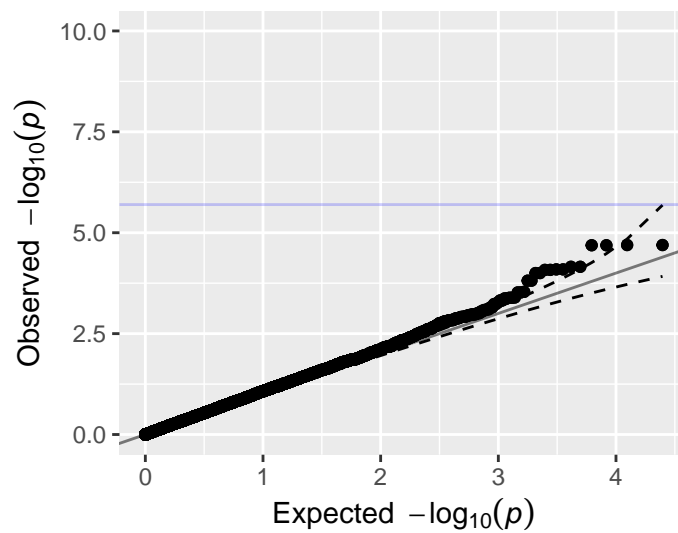

F)

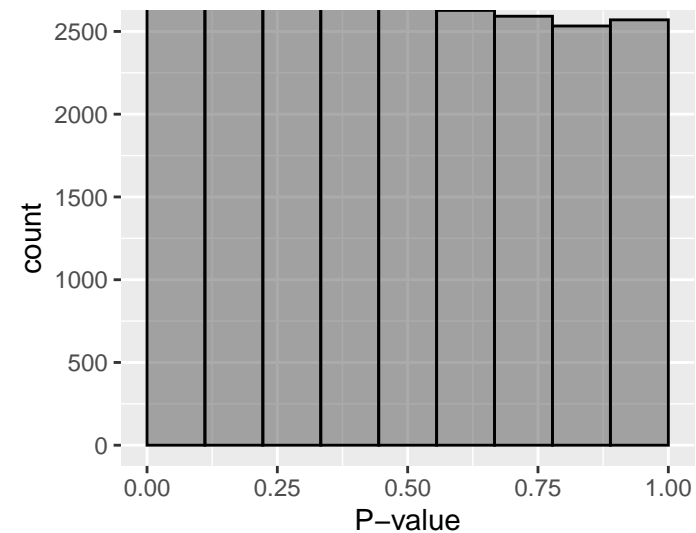

G)

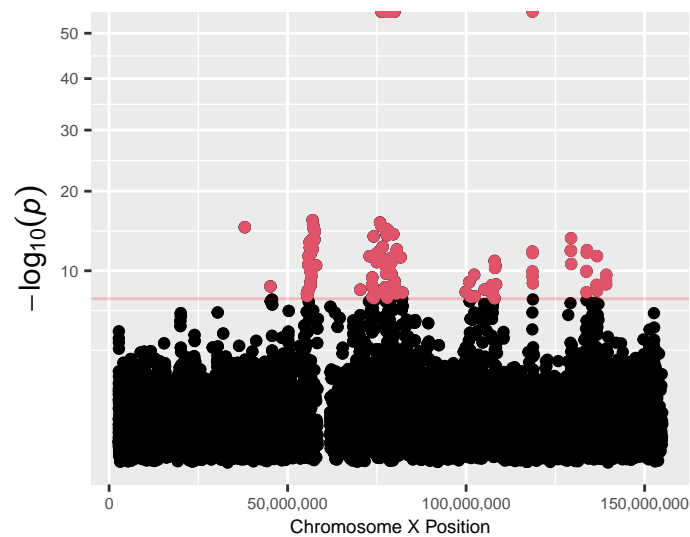H)  
gJLS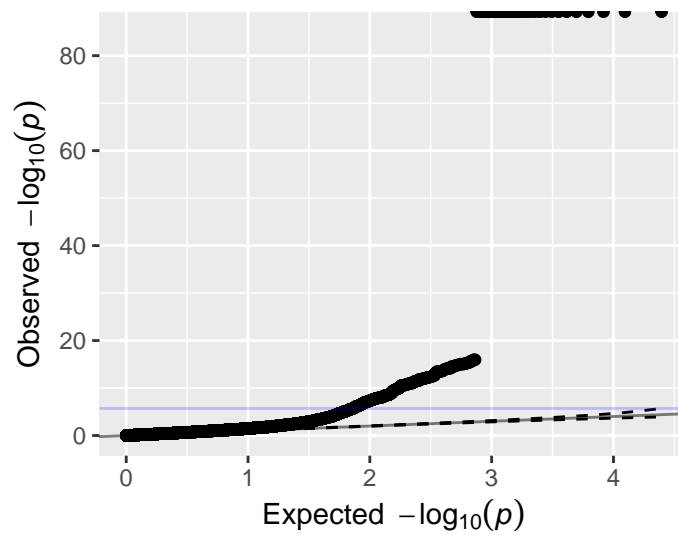

I)

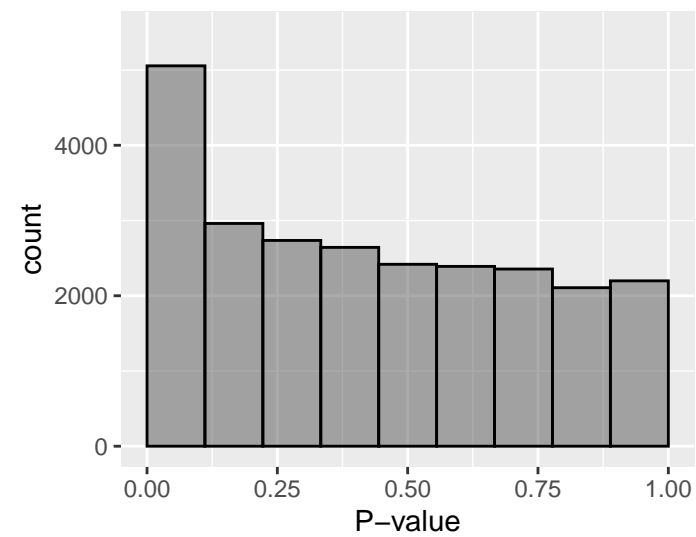

Supplement: jkac049_Supplementary_Data [file jkac049_supplementary_data.zip › Suppl/Supp.Figure_7_G3-2022-403216.pdf]

A)

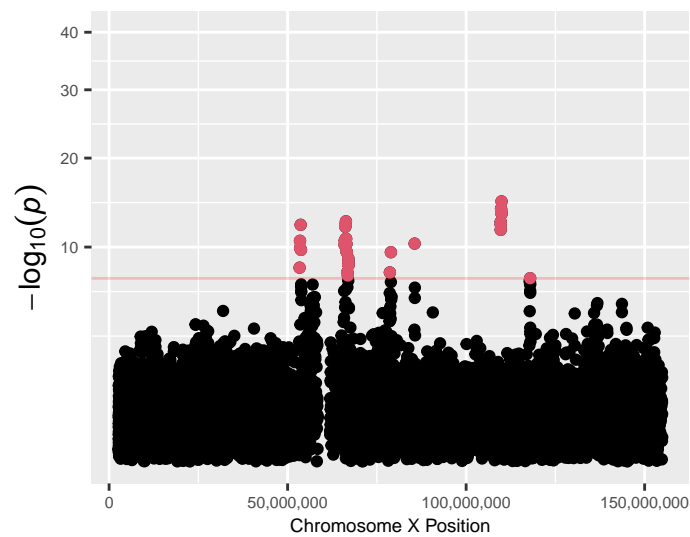B)  
Location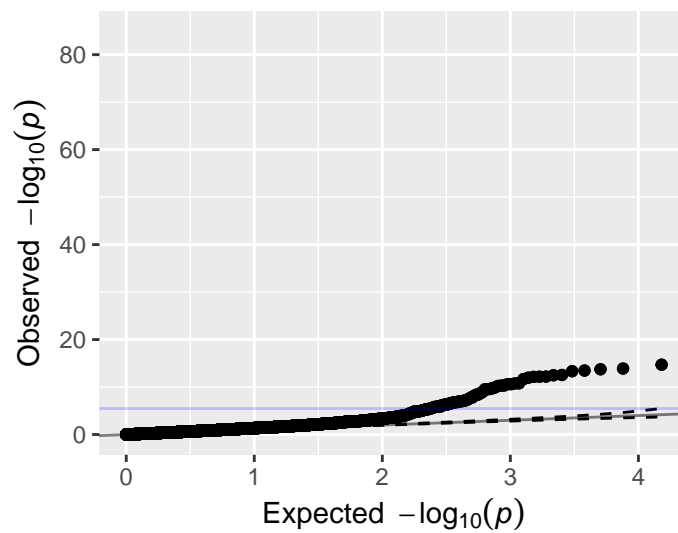

C)

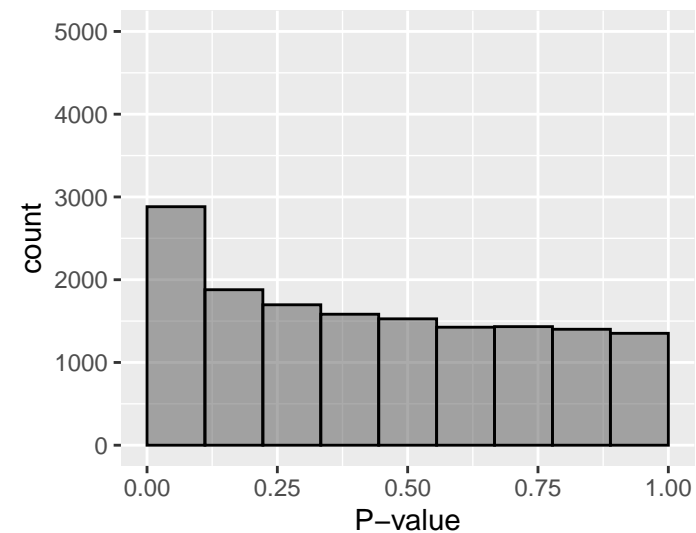

D)

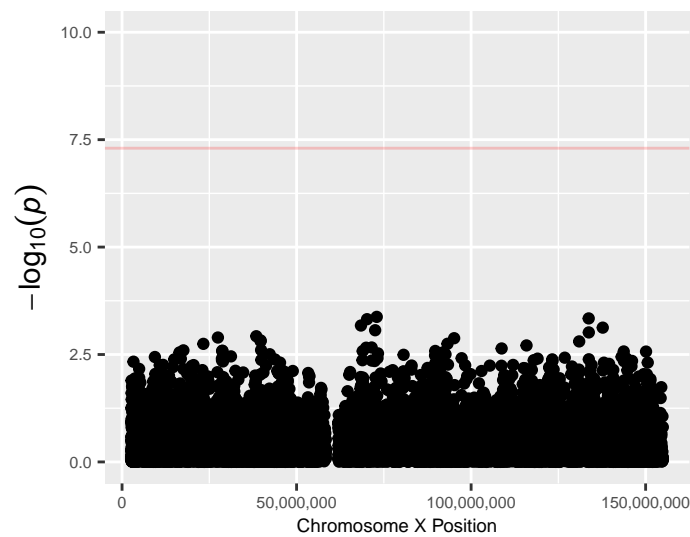E)  
Scale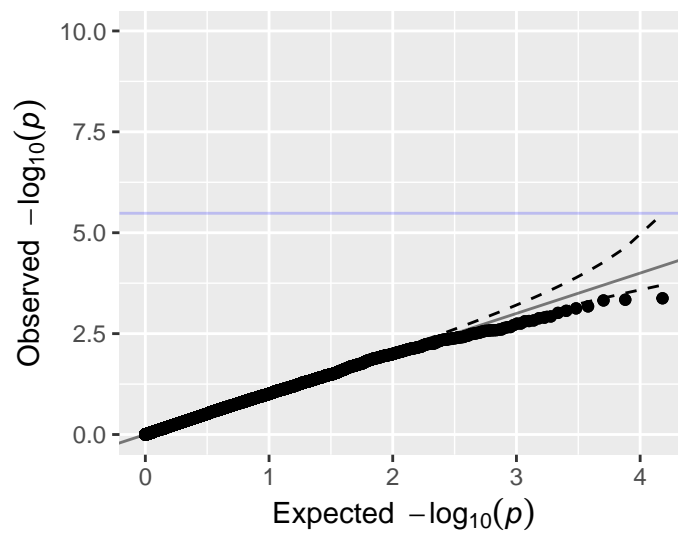

F)

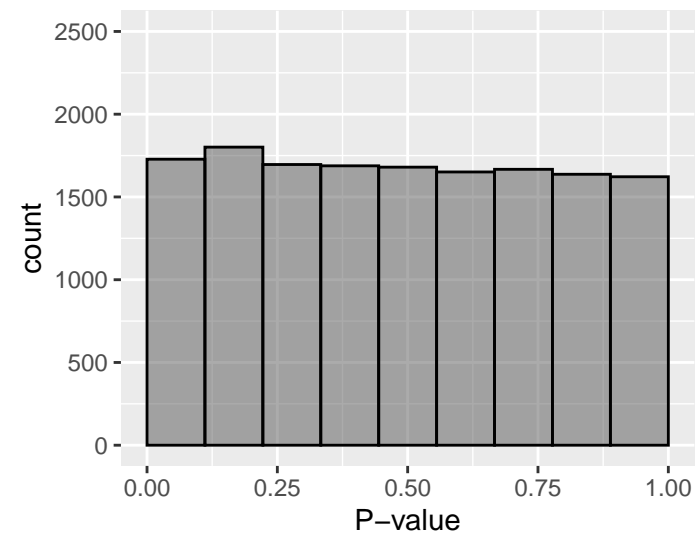

G)

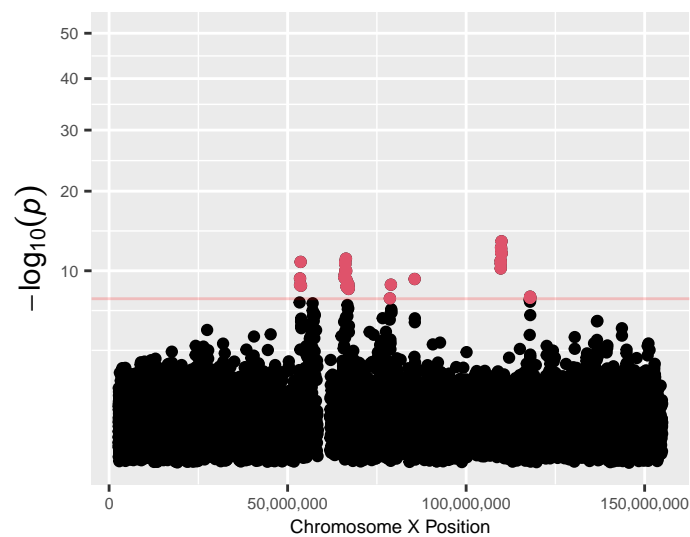H)  
gJLS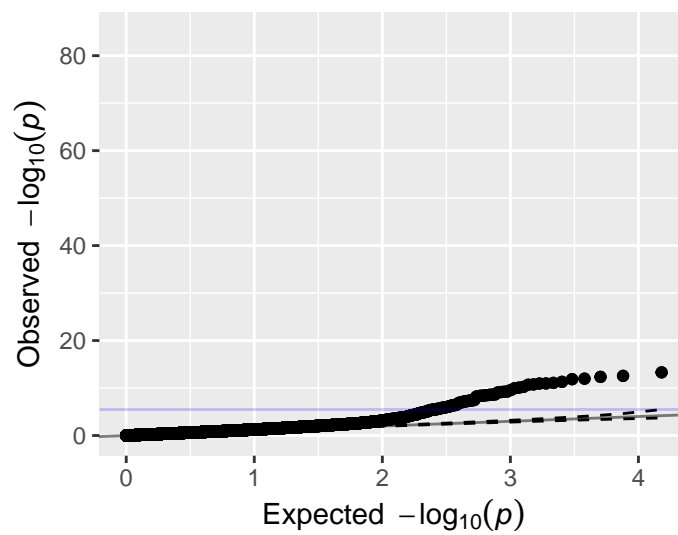

I)

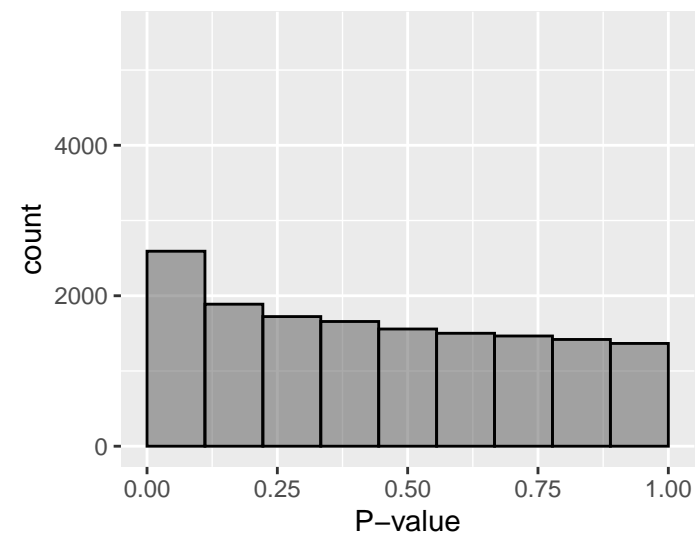

Supplement: jkac049_Supplementary_Data [file jkac049_supplementary_data.zip › Suppl/Supp.Figure_8_G3-2022-403216.pdf]

A)

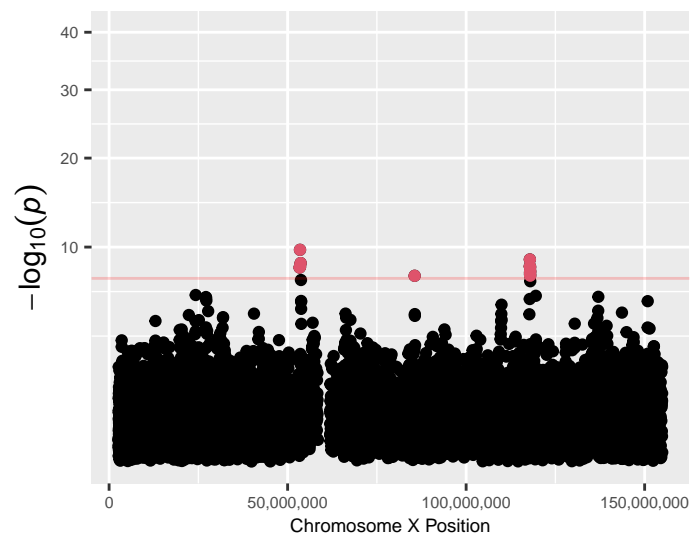B)  
Location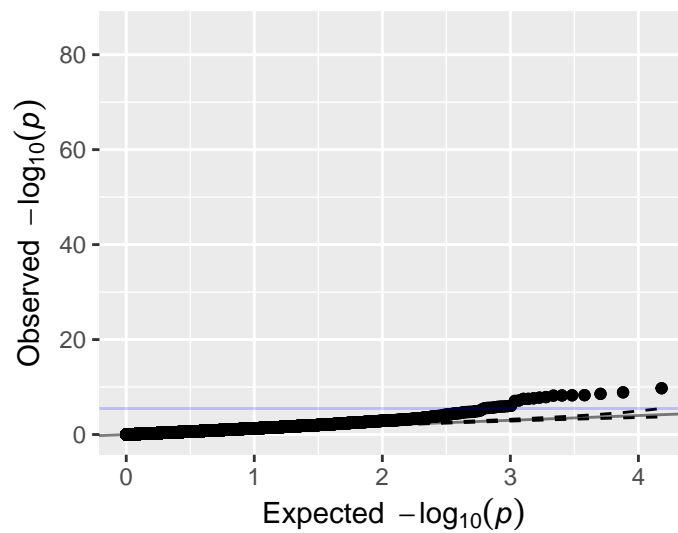

C)

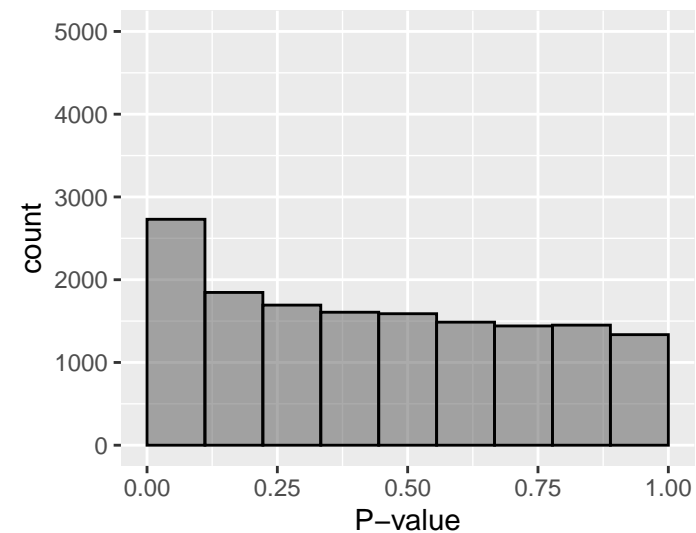

D)

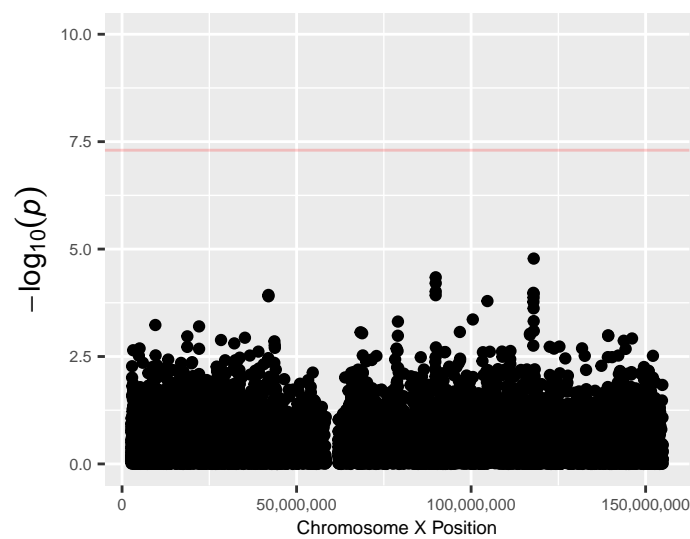E)  
Scale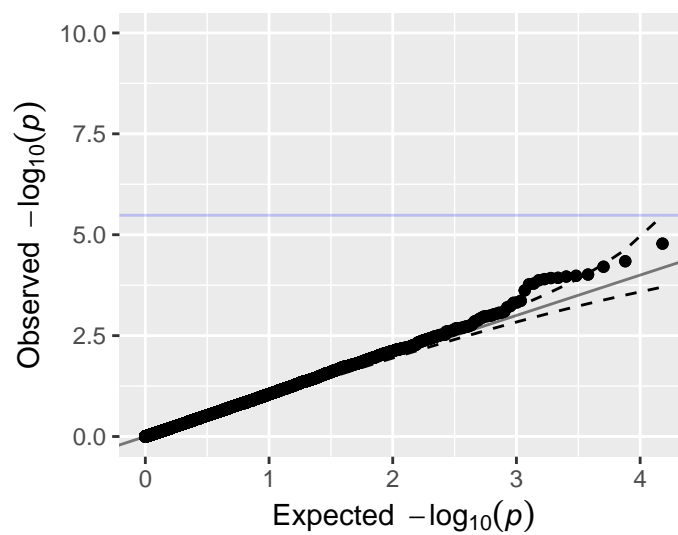

F)

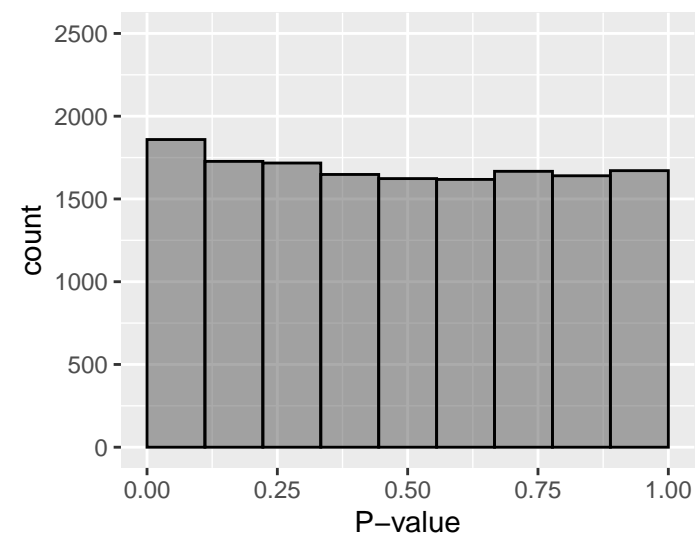

G)

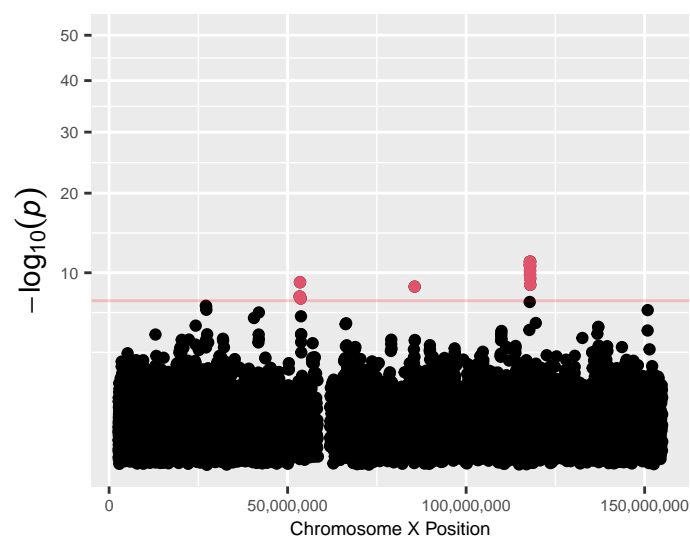H)  
gJLS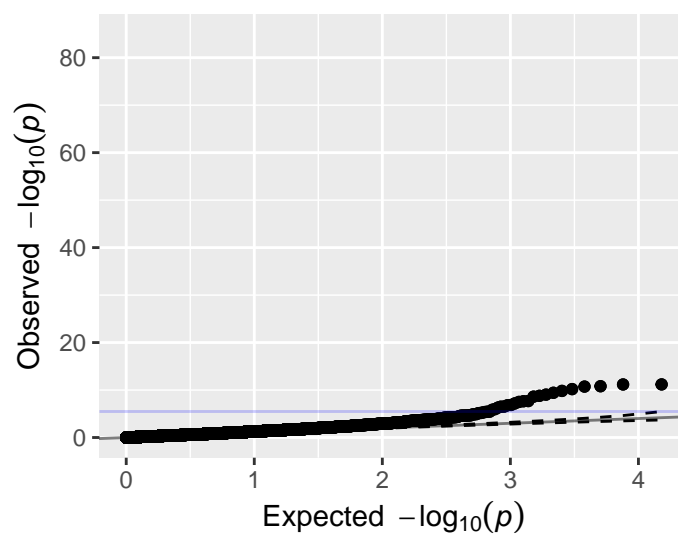

I)

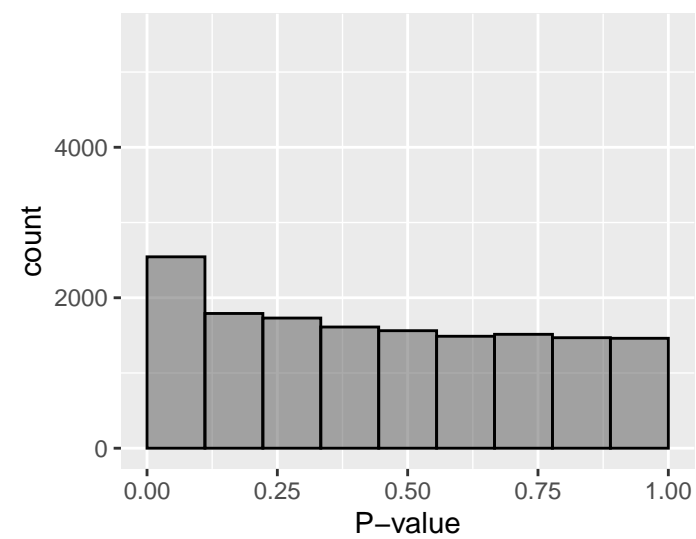

Supplement: jkac049_Supplementary_Data [file jkac049_supplementary_data.zip › Suppl/Supp.Figure_9_G3-2022-403216.pdf]
